# Classifying Autism From Crowdsourced Semistructured Speech Recordings: Machine Learning Model Comparison Study

**DOI:** 10.2196/35406

**Published:** 2022-04-14

**Authors:** Nathan A Chi, Peter Washington, Aaron Kline, Arman Husic, Cathy Hou, Chloe He, Kaitlyn Dunlap, Dennis P Wall

**Affiliations:** 1 Division of Systems Medicine Department of Pediatrics Stanford University Palo Alto, CA United States; 2 Department of Bioengineering Stanford University Stanford, CA United States; 3 Department of Computer Science Stanford University Stanford, CA United States; 4 Department of Biomedical Data Science Stanford University Stanford, CA United States; 5 Department of Psychiatry and Behavioral Sciences Stanford University Stanford, CA United States

**Keywords:** autism, mHealth, machine learning, artificial intelligence, speech, audio, child, digital data, mobile app, diagnosis

## Abstract

**Background:**

Autism spectrum disorder (ASD) is a neurodevelopmental disorder that results in altered behavior, social development, and communication patterns. In recent years, autism prevalence has tripled, with 1 in 44 children now affected. Given that traditional diagnosis is a lengthy, labor-intensive process that requires the work of trained physicians, significant attention has been given to developing systems that automatically detect autism. We work toward this goal by analyzing audio data, as prosody abnormalities are a signal of autism, with affected children displaying speech idiosyncrasies such as echolalia, monotonous intonation, atypical pitch, and irregular linguistic stress patterns.

**Objective:**

We aimed to test the ability for machine learning approaches to aid in detection of autism in self-recorded speech audio captured from children with ASD and neurotypical (NT) children in their home environments.

**Methods:**

We considered three methods to detect autism in child speech: (1) random forests trained on extracted audio features (including Mel-frequency cepstral coefficients); (2) convolutional neural networks trained on spectrograms; and (3) fine-tuned wav2vec 2.0—a state-of-the-art transformer-based speech recognition model. We trained our classifiers on our novel data set of cellphone-recorded child speech audio curated from the *Guess What?* mobile game, an app designed to crowdsource videos of children with ASD and NT children in a natural home environment.

**Results:**

The random forest classifier achieved 70% accuracy, the fine-tuned wav2vec 2.0 model achieved 77% accuracy, and the convolutional neural network achieved 79% accuracy when classifying children’s audio as either ASD or NT. We used 5-fold cross-validation to evaluate model performance.

**Conclusions:**

Our models were able to predict autism status when trained on a varied selection of home audio clips with inconsistent recording qualities, which may be more representative of real-world conditions. The results demonstrate that machine learning methods offer promise in detecting autism automatically from speech without specialized equipment.

## Introduction

Autism spectrum disorder (ASD, or autism) encompasses a spectrum of disorders characterized by delayed linguistic development, social interaction deficits, and behavioral impairments [1]. Autism prevalence has rapidly increased in recent years: according to the Centers for Disease Control and Prevention, autism rates have tripled since 2000 to 1 in 44 children in 2018 [2]. In the United States alone, over 5 million individuals are affected [3], and nearly 75 million are affected worldwide. Despite the increasing prevalence of autism, access to diagnostic resources continues to be limited, with 83.86% of all American counties not having any [4]. These nationwide inadequacies in autism resources are compounded by the lengthy nature of diagnosis. On average, the delay from the time of first consultations with health care providers to the time of diagnosis is over 2 years. Such extensive delays often cause diagnosis at a later age (usually ≥4 years old) [5], which may result in greater lifelong impacts, including a higher likelihood of psychotropic medication use, lower IQ scores, and reduced language aptitude [6,7]. Given that timely autism identification and intervention has been shown to improve treatment success and social capabilities, research has focused on its early detection [7-11].

Although symptoms vary across individuals, prosody abnormalities are among the most notable signs of autism, with multiple studies suggesting that affected children display peculiarities including echolalia, monotonous intonation, and atypical pitch and linguistic stress patterns [12-14]. Given this, an effective artificial intelligence sound classifier trained to detect speech abnormalities common in children with autism would be a valuable tool to aid autism diagnostic processes.

Prior research [15,16] investigated prosodic disorders in children with autism to varying degrees of success. Cho et al [17] developed models that achieved 76% accuracy on a dataset of recorded interviews between children and unfamiliar adults, trained on data recorded at a consistent location using a specialized biosensor device with 4 directional microphones. Similarly, Li et al [18] achieved high accuracies when training on speech data recorded with multiple wireless microphones, providing high purity recordings at a central recording location (a hospital). However, both used data collected in centralized, unfamiliar locations with high-quality recording equipment. Such research, while promising, does not accelerate the process of autism detection because it requires the use of specialized equipment and centralized recording locations to provide consistent audio quality, posing significant barriers to the widespread availability of automatic diagnosis tools. Additionally, interacting with unknown adults in foreign environments could be stressful and possibly affect the behavior of children with autism, thus leading to observations that are not generalizable to the real world.

In this work, we propose a machine learning–based approach to predict signs of autism directly from self-recorded semistructured home audio clips recording a child’s natural behavior. We use random forests, convolutional neural networks (CNNs), and fine-tuned wav2vec 2.0 models to identify differences in speech between children with autism and neurotypical (NT) controls. One strength of our approach is that our models are trained on mobile device audio recordings of varying audio quality. Therefore, unlike other studies, our approach does not necessitate specialized high-fidelity recording equipment. Additionally, we attempt to capture naturalistic speech patterns by recording children playing educational games with their parents in a low-stress home environment. Finally, our approach does not require a trained clinician to converse with the child. To our knowledge, our method is the first to aurally detect symptoms of autism in an unstructured home environment without the use of specialized audio recording devices. 

## Methods

### Data Acquisition

#### Process

**Figure 1:**
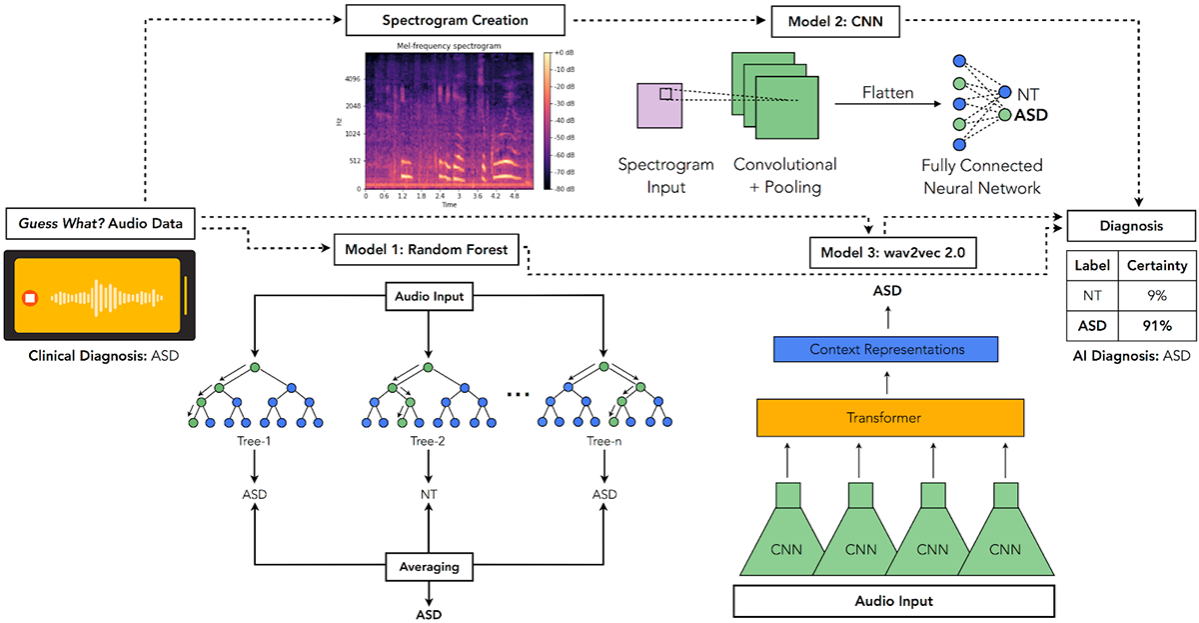
Overview of audio-based AI detection pipeline. First, the educational video game *Guess What?* crowdsources the recording of videos of NT children and children with ASD from consenting participants. Audio of children's speech is manually spliced from the videos and 3 models are trained on this audio data. The first is a random forest classifier, which uses an ensemble of independently trained decision trees. The second is a CNN. The third is a fine-tuned wav2vec 2.0 model. Model 1 takes commonly used speech recognition features as input, model 2 learns from spectrograms of the audio, and model 3 takes the raw audio data itself as input. AI: artificial intelligence; ASD: autism spectrum disorder; CNN: convolutional neural network; NT: neurotypical.

We obtained audio data of NT children and children with autism in a home environment through *Guess What?*, a mobile game designed for prosocial play and interaction at home between 2- to 8-year-old developing children and their parents [19-23] (Figure 1, “*Guess What?* Audio Data”). During a game session, parents and children choose either a charades game (acting out emotions, characters, sports, chores, or objects) or a simple quiz game (identifying colors, shapes, numbers, and word spellings). Children are directed to follow the rules of gameplay, while parents serve as game mediators. Throughout the session, parents record their children by placing their smartphones on their foreheads with the front-facing camera oriented toward the child. After each 90-second session, parents are given the option to view their child’s game session video recording and share it with our research team.

#### Distribution of Demographics

We collected a total of 77 videos of 58 children participating in gameplay, recorded in the span of 4 years from 2018 to 2021. The participants ranged in age from 3-12 years old and included 20 children with ASD (19 male and 1 female) and 38 NT children (15 male, 22 female, and 1 unspecified). The median age of the children with ASD was 5 years; the median age of the NT children was 9.5 years. Parents involved in the study consented to sharing their videos with our research team and provided their child’s age, sex, and diagnosis.

#### Advantages

This pipeline offers several benefits over traditional diagnostic workflows. Since only a smartphone is necessary, more children can be assessed for autism than through in-lab procedures, with lower costs of time and health care resource use. Through *Guess What?*, a traditionally time-intensive health care process for diagnosis could potentially be transformed into a quick and enjoyable process. Furthermore, children recorded at home may be more likely to behave in a naturalistic manner.

### Data Preprocessing

Home videos are naturally variable in quality; their data contains a number of irregularities that must be addressed prior to analysis. In particular, parents or children would often join in gameplay simultaneously, resulting in a variety of voices, sometimes overlapping with one another. This overlap of voices can complicate the isolation and extraction of the child’s voice. In order to remove adult speech, we manually sampled only child speech from each video, ensuring that each resulting clip did not include any voice other than the child’s. Each child contributed a mean of 1.32 videos and 14.7 clips, resulting in a total data set size of 850 audio clips, representing 425 ASD and 425 NT clips. The 850 clips were split into 5 folds, as shown in Table 1, in preparation for 5-fold cross-validation. When creating the folds, we included the restriction that all clips spliced from a given child’s video had to be included in the same fold to prevent models from learning from child-specific recording idiosyncrasies, including environmental background noise and audio quality.

**Table 1:** Distribution of 850 audio clips across 5 folds. Each of the 3 models was trained on the same distribution of clips with 5-fold cross-validation.

Group	Fold 0	Fold 1	Fold 2	Fold 3	Fold 4
Neurotypical	87	87	81	83	87
Autism spectrum disorder	87	87	81	83	87

### Classifiers

We investigated 3 machine learning methods to predict autism from audio, each represented in Figure 1.

#### Random Forest

We trained random forests on a set of audio features (Mel-frequency cepstral coefficients, chroma features, root mean square, spectral centroids, spectral bandwidths, spectral rolloff, and zero-crossing rates) typically used in traditional signal processing speech recognition. We also tried training other models (including logistic regression, Gaussian Naive Bayes, and AdaBoosting models), which did not perform as well. We implemented the random forest model in scikit-learn and used the following manually chosen hyperparameters: max_depth_=20,000, *n*_estimators_=56, max_features_=15, min_samples split_=10, min_samples leaf_=20, min_weight fraction leaf_=0.1.

#### CNN Model

**Figure 2:**
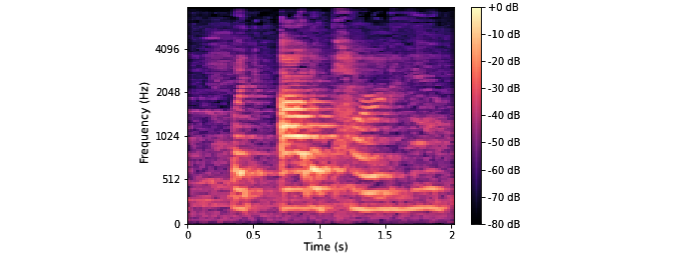
Mel-frequency spectrogram for a neurotypical child speech segment, spliced from a *Guess What?* gameplay video. This spectrogram was one of 850 used to train the convolutional neural network model with 8 million parameters, which yielded the highest accuracy of the 3 best-performing models.

**Figure 3:**
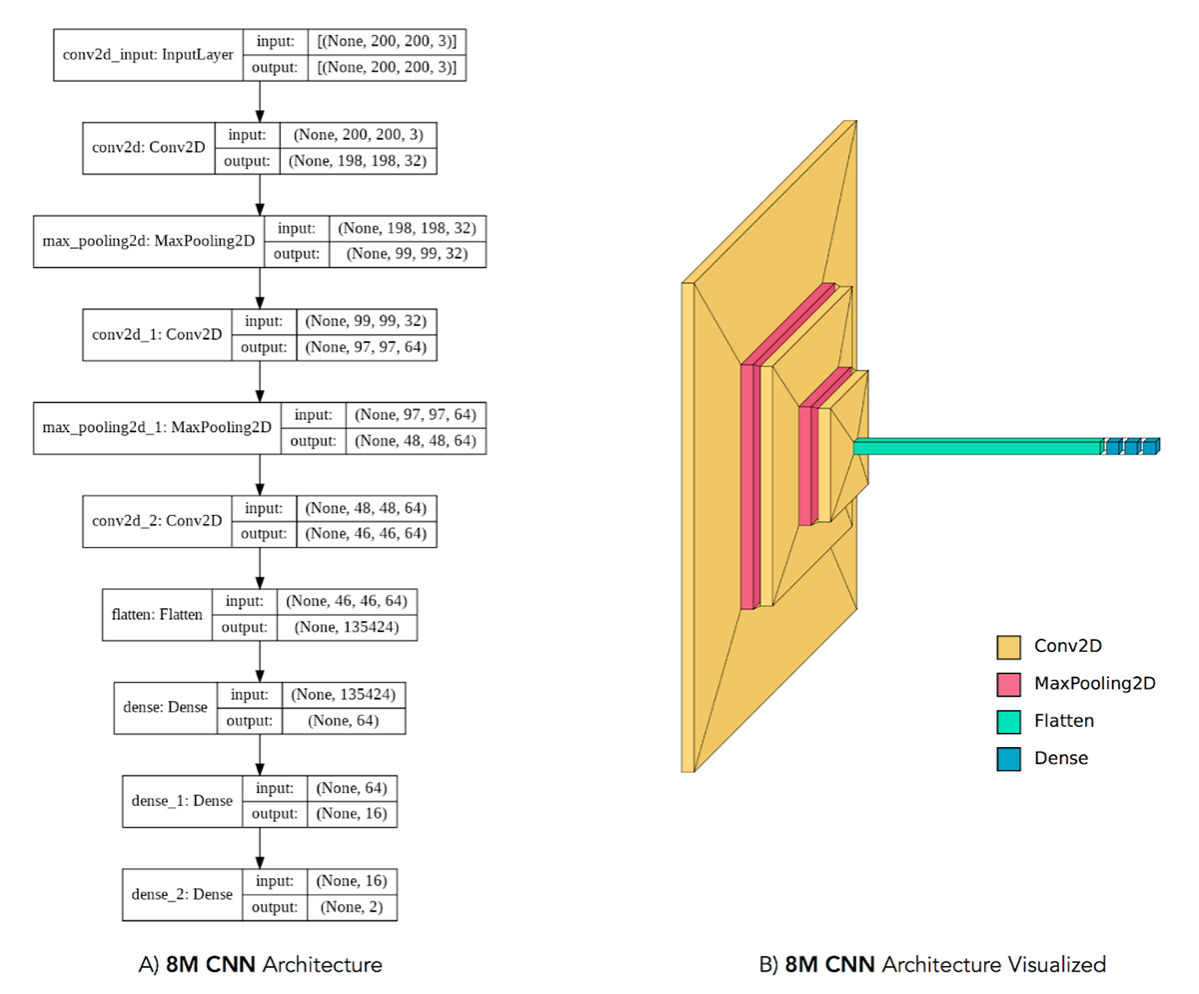
(A) and (B) represent the same 8M CNN model architecture. This architecture performed best out of all of our tested architectures, including a fine-tuned Inception v3 model. (B) was in part created with the Python package Visualkeras. 8M CNN: convolutional neural network with 8 million parameters.

We trained a CNN using spectrograms of our data as input [24,25]. Our spectrograms were synthesized via the Python package Librosa. Figure 2 shows an example of the spectrograms used to train the CNN. The CNN, represented in Figure 3, consists of 9 layers each with alternating convolution and max pooling layers, as well as 3 dense layers with a L2 regularization penalty of 0.01. We investigated both training a small CNN (~8 million parameters) from scratch and fine-tuning the image recognition model Inception v3 (with ~33 million parameters) trained on ImageNet [26]. However, our CNN model with 8 million parameters ultimately performed slightly better than the transfer learning approach, likely due to the irrelevance of ImageNet features to spectrograms. Our final CNN model, which we train for 15 epochs (until training performance stopped improving), has 8,724,594 parameters.

#### Wav2vec 2.0

We fine-tuned wav2vec 2.0, a state-of-the-art transformer model pretrained on a self-supervised audio denoising task [27]. Although wav2vec 2.0 is typically used for speech-to-text decoding, prior work [28] has demonstrated its utility for suprasegmental tasks such as emotion prediction. We used the facebook/wav2vec2-base variant and fine-tuned for 264 steps. The final model has 95 million parameters.

#### Summary

For each method, we trained and evaluated using 5-fold cross-validation. We ensured that clips from a child are maintained in one fold to prevent the model from artificially performing better by learning user recording idiosyncrasies (eg, background noise). For each fold, we saved the weights for the highest performing model after training and reported mean accuracy (with threshold 0.5), precision, recall, *F*_1_ score, and area under the receiver operating characteristic curve (AUROC), averaged over the 5 folds.

## Results

Of our models, the best-performing model was the CNN model with 8 million parameters, achieving 79.3% accuracy, 80.4% precision, 79.3% recall, 79.0% *F*_1_ score, and a mean AUROC score of 0.822 (Table 2). Our wav2vec 2.0 model performed comparably with our best CNN, achieving 76.9% accuracy, 78.2% precision, 74.6% recall, and 76.8% *F*_1_ score, and a mean AUROC score of 0.815. On the other hand, our highest performing lightweight machine learning model (random forest) performed somewhat worse than the other 2 models, with 69.7% accuracy, 68.7% precision, 74.4% recall, 69.4% *F*_1_ score, and a mean AUROC score of 0.740.

**Figure 4:**
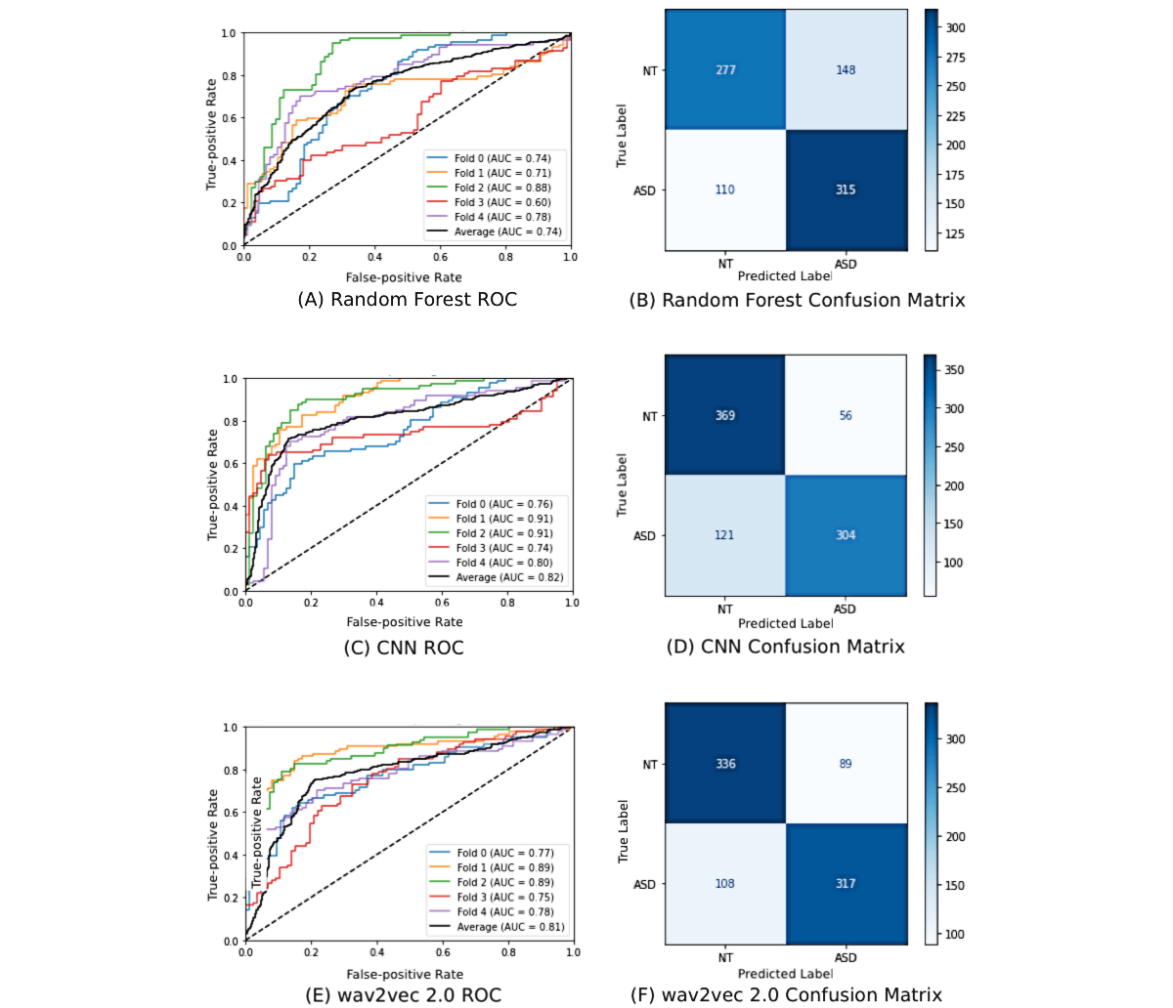
(A) ROC curve for random forest model. (B) Confusion matrix for random forest model. (C) ROC curve for 8M CNN. (D) Confusion matrix for CNN. (E) ROC curve for wav2vec 2.0 model. (F) Confusion matrix for wav2vec 2.0 model. All models were tested and trained on the *Guess What?* audio data set, composed of child speech segments taken from educational gameplay videos. 8M CNN: convolutional neural network with 8 million parameters; ASD: autism spectrum disorder; AUC: area under the curve; NT: neurotypical; ROC: receiver operating characteristic.

Our receiver operating characteristic (ROC) curves for the top 3 highest performing models of each category are included in Figure 4, panels A, C, and E. In each figure, ROC curves for each individual fold and the mean curve are reported. One point of interest is that each figure has variation in area under the curve (AUC) values between folds to some degree. Moreover, these variation trends are similar between models: for instance, each model appears to perform well on fold 2 while performing relatively poorly on fold 3. This suggests that the data in each fold may be too limited, resulting in folds that have differences in content that cause varying model performance from fold to fold. This disparity between AUC values is the greatest in Figure 4A, perhaps explainable by the random forest classifier’s small size and lightweight traits. The wav2vec model (Figure 4E) has the most unvarying results, implying that it is better at consistently performing well at classifying unseen data than either of the other two models. This is expected, given that the wav2vec model contains far more parameters than either of the other two models and is more robust.

In Figure 4, panels B, D, and F, we provide confusion matrices for all 3 highest performing models. Figure 4D and Figure 4F show that both the CNN and wav2vec models have relatively few false positive predictions, while Figure 4B shows that the random forest classifier has a relatively large number of false positive predictions. All models have similar false negative prediction rates.

**Table 2:** Performances on *Guess What?* data set. Results are reported with standard deviation over 5 different runs for each model.

Model	Accuracy, mean (SD)	Precision, mean (SD)	Recall, mean (SD)	*F*_1_ score, mean (SD)	AUROC^a^, mean (SD)
Random forest	0.697 (0.013)	0.687 (0.010)	0.744 (0.247)	0.694 (0.013)	0.740 (0.09)
Convolutional neural network	0.793 (0.013)	0.804 (0.014)	0.793 (0.014)	0.790 (0.014)	0.822 (0.010)
Wav2vec 2.0	0.769 (0.005)	0.782 (0.021)	0.746 (0.031)	0.768 (0.006)	0.815 (0.077)

^a^AUROC: area under the receiver operating characteristic curve.

## Discussion

### Principal Results

We trained multiple models to detect autism from our novel data set of audio recordings curated from the educational video game *Guess What?* We presented a set of systems that classify audio recordings by autism status and demonstrated that both CNNs and state-of-the-art speech recognition models are capable of attaining robust performance on this task, with lightweight statistical classifiers still achieving reasonable results.

### Privacy

One consideration for any recorded audio medical diagnosis is privacy [29-31], which is particularly important for studies involving commonly stigmatized disorders like autism [32]. We note that since our proposed models are relatively lightweight, they could feasibly be deployed at home on mobile devices, allowing for private offline symptom detection as well as privacy-preserving federated learning approaches [33]. Prior work investigated using federated learning techniques to preserve privacy while boosting model performance on a functional magnetic resonance imaging classifier task; a similar framework might be feasible for autism diagnosis, affording a greater degree of privacy for parents who wish for a diagnostic signal but hesitate to share videos with strangers [34].

### Limitations

One limitation of our approach is the relative imbalances in the gender distribution of children who comprised our speech data set. Our data set included a split between 95% males with ASD and 5% females with ASD for autistic speech segments, as well as a 39% NT male, 58% NT female, and 3% NT unknown gender split for NT speech segments. Our data set had a sizable imbalance in terms of the relative proportion of males and females with ASD represented. Although some imbalance is to be expected due to the naturally skewed autism sex ratio, our imbalance was larger than the observed real-world 4:1 to 3:1 male-to-female incidence ratio, which would result in a data set containing an 80%-75% male and 20%-25% female split for ASD segments [35,36]. Therefore, despite being closer to replicating actual conditions than prior work, our data set may still not be completely representative of real-world conditions. Additionally, while we require parents to disclose their child’s clinical diagnosis by choosing from options not widely known to those who have not received a clinical evaluation, these labels are self-reported and thus unverified.

Another limitation of our work is that we evaluated on a relatively small data set. Additionally, manually splicing videos to isolate child voices is a time-intensive process that may not be scalable to larger data sets. The alternative—automatically isolating voices through blind signal separation—is an exceptionally challenging task [37,38]. However, it poses a potential area of interest and is possibly a necessary hurdle to overcome to develop widely available and consistently effective autism machine learning diagnosis resources.

### Future Work

One strength of our approach is the relatively small amount of data required to train the model. Our models were trained on clips spliced from a total of 115.5 minutes of audio yet still yielded relatively accurate results, implying that training on more data may improve performance.

Therefore, future directions include testing our models’ performance with additional data from a wider selection of both children with autism and NT children. One particular area of interest may be wearable devices such as Google Glass [39,40]; previous work [41-44] investigated delivering actionable, unobtrusive social cues through wearables. Such approaches have been demonstrated to improve socialization among children with ASD [10,45], suggesting that they could also be used to collect naturalistic data similar to this experiment in an unobtrusive way.

Another area of interest for future work may be examining the possibility of leveraging a distributed workforce of humans for extracting audio-related features to bolster detection accuracy. Previous work examined the use of crowdsourced annotations for autism, indicating that similar approaches could perhaps be applied through audio [31,46-51]. Audio feature extraction combined with other autism classifiers could be used to create an explainable diagnostic system [52-64] fit for mobile devices [60]. Previous work investigated using such classifiers to detect autism or approach autism-related tasks like identifying emotion to improve socialization skills; combining computer vision–based quantification of relevant areas of interest, including hand stimming [58], upper limb movement [63], and eye contact [62,64], could possibly result in interpretable models.

### Conclusions

Use of automatic audio classification could help to accelerate and improve the accuracy and objectivity of the lengthy diagnosis process for autism. Our models were able to predict autism status by training on a varied selection of home audio clips with inconsistent recording quality, which may be more representative of real-world conditions. Overall, our work suggests a promising future for at-home detection of ASD.

## Abbreviations

ASD: autism spectrum disorder
AUC: area under the curve
AUROC: area under the receiver operating characteristic curve
CNN: convolutional neural network
NT: neurotypical
ROC: receiver operating characteristic
